# Concentration of survivin in children with oligo- and polyarticular juvenile idiopathic arthritis (JIA): diagnostic and prognostic value—a single-center study

**DOI:** 10.1186/s13075-021-02424-y

**Published:** 2021-01-26

**Authors:** Joanna Lipinska, Marcin Kaszkowiak, Beata Malachowska, Joanna Swidrowska-Jaros, Elzbieta Smolewska

**Affiliations:** 1 Department of Pediatric Cardiology and Rheumatology, Medical University of Lodz, 36/50 Sporna St., 91-738 Lodz, Poland; 2 Outpatient Department of Pediatric Rheumatology, Maria Konopnicka’ Memorial Hospital, Lodz, Poland; 3 Department of Biostatistics and Translational Medicine, Medical University of Lodz, Lodz, Poland; 4 Department of Radiation Oncology, Albert Einstein College of Medicine, Bronx, NY USA

**Keywords:** Survivin, Juvenile idiopathic arthritis, Disease activity, Biomarkers, Autoantibodies, Prognosis, Diagnosis, Biological treatment, Radiological damage, Synovitis

## Abstract

**Aim:**

The goal of the study was to assess the diagnostic and prognostic utility of survivin in patients with juvenile idiopathic arthritis (JIA).

**Methods:**

Seventy children with JIA—59 newly diagnosed and 11 biologically treated (46 girls and 17 boys) aged 1.5–18 years and 29 healthy children as a control group, appropriately matched in terms of sex and age, were included in the study. The disease activity was established on the basis of the JADAS-27 criteria. The concentration of survivin was assessed by an ELISA test in serum and also 18 matched synovial fluid samples collected from patients with JIA.

**Results:**

Children with JIA were divided according to the subtype of the JIA. In 65.7% of patients, oligoarthritis was diagnosed. The largest group comprised children of low disease activity (62.9%) according to JADAS-27. The serum concentration of survivin was significantly higher in children with JIA compared to the controls (*p* < 0.001). The concentration of survivin was higher among patients positive for anti-cyclic citrullinated peptide autoantibodies (ACPA) (*p* = 0.001). In all synovial fluid samples, the concentration of survivin was higher than in matched serum (*p* = 0.003). Serum survivin concentration was not significantly associated with radiological damage status or active synovitis assessed by joint ultrasonography. Survivin level was not significantly associated with disease duration time or treatment with TNF-α inhibitors in DMARD’s non-responders.

**Conclusions:**

Survivin should be considered as a biomarker of joint inflammation helpful in the diagnosis of oligo- and polyarticular JIA and probably not dependent on treatment with TNF-α inhibitors.

## Introduction

Juvenile idiopathic arthritis (JIA) is a heterogeneous group of diseases which encompasses all forms of arthritis of unknown etiology lasting for at least 6 weeks and with onset before the age of 16 [1, 2].

Due to lacking pathognomonic features, the diagnosis of JIA is made by exclusion of all possible causes of chronic arthritis in childhood [3, 4]. The International League of Associations for Rheumatology (ILAR) has defined seven subtypes of JIA [2]. While there are shared genetic and immunologic features between JIA and rheumatoid arthritis (RA) in adults, only a small subset of JIA patients with polyarticular disease and a positive rheumatoid factor (RF) clinically resembles adult RA patients [5]. Advances in our understanding of the JIA pathogenesis over the last two decades have revolutionized therapy, reduced morbidity, and improved quality of life for those affected [3, 4]. Various autoantibodies have been associated with JIA, including anti-nuclear antibodies (ANA), RF, anti-citrullinated protein autoantibodies (ACPA), and others. Although the ANA test is not used to diagnose JIA, it is of high prognostic value with respect to the risk of uveitis. ANA positivity among the JIA subtypes is the highest in patients with oligoarticular JIA (up to 70%) and is particularly more prevalent in young, female patients. The prevalence of RF in patients with JIA is very low (< 5%), and it confers a worse prognosis. In particular, RF-positive polyarticular patients are at higher risk of a more aggressive disease course and bone erosion [3, 4]. The identification of ACPA, highly specific for adult RA, was a milestone for adult rheumatology. ACPA have been shown to predict future risk for developing RA in otherwise healthy individuals. However, as with RF, the sensitivity of ACPA for detecting JIA is low. But in RF-positive patients with polyarticular JIA, these autoantibodies are highly specific and predict a more severe disease course [6]. ACPA-positive children with JIA are recommended for earlier and more aggressive therapy. Nevertheless, the diagnosis of JIA still depends mainly on clinical characteristics, imaging examination, and exclusion of other, more common causes of persistent arthritis with low serological support [3–6]. Therefore, it is necessary to establish alternate methods or discover new biomarkers to further improve precise JIA diagnosis at the early stage of the disease.

Survivin is an anti-apoptotic oncoprotein, known as a tissue marker of cancer. During recent years, the role of survivin in non-malignant cells was intensively explored. Survivin has been shown essential for the differentiation, growth, and regeneration of healthy tissues. Due to survivin roles in apoptosis and proliferation, it plays important roles in the pathogenesis of autoimmune diseases [7]. At the preclinical phase of RA, high levels of survivin correlate with cytokines and anticipate the formation of aggressive Th1 and Th17 cells. In patients after RA diagnosis, survivin predicts joint destructive course of the disease and resistance to anti-rheumatic treatment [8–10]. Survivin has been suggested as a predictive marker of a severe course of adult RA and could be used for preclinical recognition of the disease. Survivin-positive patients have poor outcomes if treated with methotrexate (MTX) monotherapy. A decrease in serum survivin concentration is associated with a better clinical response to treatment [11]. Despite therapy advances in JIA, patients can achieve only symptoms alleviation but cannot be completely cured. Therefore, exploring the pathogenesis of the rheumatoid process is of high importance for developing precise, personalized treatments and new drug targets.

Due to great disease heterogeneity and low number of patients, studying JIA pathogenesis is much more challenging than adult RA. Thus, much of our knowledge about the role of RF, ACPA, and other biomarkers in inflammatory arthritis is derived from the adult literature. This is clearly a limitation, since these studies only pertain to a small subset of JIA patients overall, specifically polyarticular children RF-positive.

Taking advantage of the recent reports in adults with RA, we designed this study aiming to validate the diagnostic and prognostic utility of survivin in patients with JIA.

## Material and methods

### Patients

Seventy children diagnosed with JIA before 16 years old according to the 2001 Edmonton ILAR classification criteria [12, Petty 2001] (51 girls and 19 boys) aged 1.5–17.5 years (Me 10.25 years) were included in the study. Patients with JIA biologically treated were significantly older (Me 17.0 vs 9.0 years) and had a longer disease duration time (Me 84.0 vs 3.0 months) than the biologically naive group with JIA. Additionally, 29 healthy children were recruited as a control group and were appropriately matched in terms of sex (20 girls and 9 boys) and age (2.0–16.5, median 12.1) to the study group. Children with JIA were divided according to the subtype of the disease. The majority of the study group—65.7%—was diagnosed with oligoarthritis, and 32.9% of children had polyarticular subtype of the JIA. In the study group, there was only one child with systemic disease. The type of onset was defined according to the ILAR criteria (2001) [12]. The activity of the disease was established on the basis of the 27-joint Juvenile Arthritis Disease Activity Score (JADAS-27) [13]. Low, intermediate, and high stages of the disease activity have been distinguished accordingly.

Patient characteristics were presented in Table 1.

**Table 1:** Characteristics of the study group

JIA	All patients (*N* = 70, 100%)	Newly diagnosed (*N* = 59, 84.3%)	Biologically treated (*N* = 11, 15.7%)	*p* value
**Sex**				
Girls	51 (72.9%)	43 (72.9%)	8 (72.7%)	
Boys	19 (27.1%)	16 (27.1%)	3 (27.3%)	1.000
**Age** [years]	10.25 (6.5–15.5)	9.0 (5.5–14.5)	17.0 (14.5–17.5)	< 0.001
**Age at onset** [years]	7.00 (3.5–12.5)	7.5 (4.0–13.5)	4.0 (2.0–10.0)	0.095
**Disease duration time** [months]	6.0 (1.5–36.0)	3.0 (1.0–18.0)	84.0 (72.0–132.0)	< 0.001
**Disease onset type**				
Oligoarthritis	46 (65.7%)	41 (69.5%)	5 (45.5%)	
Polyarthritis	23 (32.9%)	18 (30.5%)	5 (45.5%)	0.073
Systemic	1 (1.4%)	0 (0%)	1 (9%)	
**Disease activity (JADAS-27)**				
Low/medium/high	44 (62.9%)	36 (61.0%)	8 (72.7%)	1.000
	17 (24.3%)	14 (23.7%)	3 (27.3%)	
	9 (12.86%)	9 (15.3%)	0 (0%)	
WBC [G/L]	7.25 (5.6–9.6)	7.2 (5.5–10.7)	7.7 (6.2–9.2)	0.675
PLT [G/L]	328.0 (254.0–420.0)	348.0 (269.0–425.0)	228.0 (206.0–349.0)	0.006
ESR [mm/h]	13.0 (7.0–25.0)	15.0 (8.0–35.0)	6.0 (3.0–15.0)	0.007
CRP [*N* < 5.0 mg/dl]	1.6 (0.4–8.9)	2.0 (0.4–11.8)	0.4 (0.2–2.7)	0.057
ACPA (anti-CCP) > 5 mg/dl	17 (24.3%)	13 (22.0%)	4 (36.4%)	0.443
ANA ≥ 1:160	33 (47.1%)	32 (54.2%)	1 (9%)	0.007
RF > 14 IU	9 (12.9%)	5 (8.5%)	4 (36.4%)	0.029
**Hands X-ray**	Stage 1–49 (70.0%)	44 (74.6%)	5 (45.5%)	0.099
	Stage 2–14 (20.0%)	10 (16.9%)	4 (36.4%)	
	Stage 3–7 (10.0%)	5 (8.5%)	2 (18.2%)	
**Joint US - PDUS**	Grade 0–11 (15.7%)	5 (8.5%)	6 (54.5%)	< 0.001
	Grade 1–20 (28.6%)	15 (25.4%)	5 (45.5%)	
	Grade 2–20 (28.6%)	20 (33.9%)	0 (0%)	
	Grade 3–19 (27.1%)	19 (32.2%)	0 (0%)	

The main group of children with JIA (59/70–84.3%) was biologically naive and had not been treated with disease-modifying anti-rheumatic drugs (DMARDs) yet. However, most of them were occasionally taking non-steroid anti-inflammatory drugs (40/59–67.7%). Additionally, a group of 11/70 (15.7%) patients with JIA that had been already treated at the time of inclusion into the study were recruited. All of them (11) have been taking DMARDs (8, methotrexate; 3, methotrexate with sulfasalazine) and TNF-α (tumor necrosis factor-α) inhibitors (3, adalimumab; 8, etanercept). Seven out of these eleven patients (7/11–63.6%) had a history of intraarticular or systemic glucocorticoids (methylprednisolone), and all 11 were occasionally taking non-steroid anti-inflammatory drugs.

The study protocol was approved by the regional Medical University of Lodz, Poland Ethical Committee [No RNN/58/13/KB].

At enrollment, all patients and their parents gave their written informed consent to participate in the study.

## Methods

Serum samples were obtained simultaneously with routine laboratory examinations, including red blood cells (RBC), white blood cells (WBC), and platelets counts (PLT), as well as erythrocyte sedimentation ratio (ESR) (cutoff value < 12 mm/h) or C-reactive protein (CRP) (cutoff value < 5 mg/L). ESR was assessed by the Westergren method and CRP level—using the immunoturbidimetric method. Additionally, ACPA, ANA, and RF were routinely measured using standard methods.

Synovial fluid, if available, was obtained during diagnostic or/and therapeutic puncture of the swollen joint.

### Measurements of serum and synovial fluid survivin

Serum and synovial fluid samples were centrifuged and stored at − 80 °C. The concentration of survivin was determined by a sandwich enzyme-linked immunoassay (ELISA test) in the serum and 18 matched synovial fluid samples of patients with JIA and in the sera of children from the control group, by commercially available kit (rabbit anti-human survivin; R&D, no DSV00, Lille, France). Synovial fluid samples, if available, were collected during diagnostic or/and therapeutic puncture of the swollen knees of children with JIA. The sensitivity of the assay was 4.45 pg/ml, with the cutoff at 9.96 pg/ml. The intra-assay precision for serum is 4.5–5.5%, and inter-assay precision is 5.7–9.5%. All serum and synovial fluid samples were tested twice.

### Joint ultrasonography and hands’ X-ray

Affected joint ultrasonography assessment was performed at the time of JIA diagnosis, by a clinician experienced in musculoskeletal ultrasonography. Synovitis detected by ultrasonography was graded as mild, moderate, or severe (score from 0 to 3). Power Doppler ultrasonographic signal (PDUS) was scored on a semiquantitative 4-grade scale: 0 = no signs of vascularization, 1 = mild (presence of single/vessel dots), 2 = moderate (presence of confluent vessel dots in less than half of the synovial area), and 3 = marked (presence of confluent vessel dots in more than half of the synovial area) (Table 1) [14].

A Philips CX50 CompactXtreme ultrasound system and a 5–12-MHz linear transducer were used in this study (Amsterdam, The Netherlands).

Conventional plain film radiographs of both hands and wrists of all children with JIA included in the study were obtained. The radiographs were scored using the Steinbrocker assessment method, with a global damage score to the hands and wrists on a 4-point scale from I (minimal damage) to IV (severe damage) as previously described (Table 1) [15].

#### Statistical analysis

Continuous data were presented as median with interquartile range, and categorical data were presented as number with respective percentage. The differences for continuous variables were tested with the Kruskal-Wallis ANOVA, Wilcoxon signed-rank test, or *U* Mann-Whitney rank sum test. Nominal variables were analyzed using the *χ*^2^ test or Fisher’s test when appropriate. Correlation analyses were performed with the Spearman rank test. Additionally, the receiving operating characteristic (ROC) curve was used for determining the best cutoff value for the survivin concentration in JIA diagnosis. Sensitivity and specificity together with 95% confidence intervals (CI) were calculated for selected cutoff value with http://vassarstats.net/online tool. The area under the curve (AUC) was calculated to evaluate the diagnostic value of survivin. All tests were two-tailed and performed at the 0.05 level of significance. The statistical analyses were carried out with the Statistica 13.1 (Tibco, Tulsa, OK, USA) and VassarStats tool (http://vassarstats.net/).

## Results

Majority of the newly diagnosed children with JIA comprised with oligoarticular subtype of JIA (41/59–69.5%), whereas 18/59 (30.5%) patients represented polyarticular JIA onset type and none among newly diagnosed had systemic JIA. In the whole study group, the largest cohort of children with JIA had low disease activity (44/70–62.9%) established on the basis of the JADAS-27 criteria. There were 17/70 (24.3%) children with JIA and medium and only 9/70 (12.9%) with high disease activity.

**Fig. 1:**
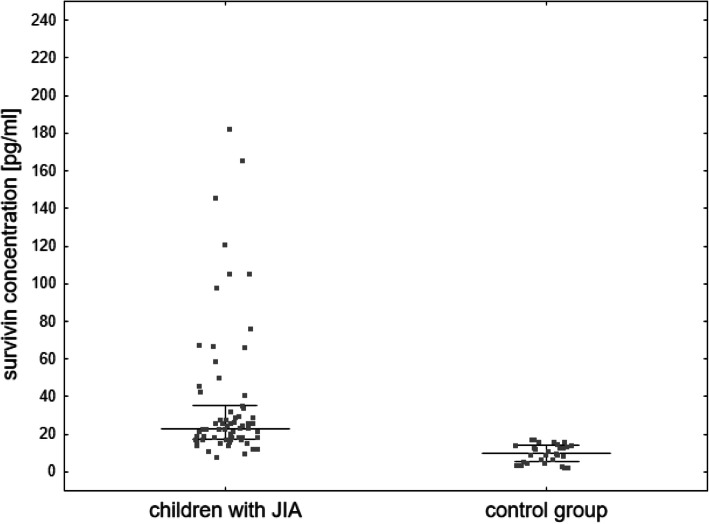
Serum survivin concentration (median and IQR) in children with JIA and in the control group. *Patient with value 537.82 pg/ml was not shown in the graph but contributed to median and IQR calculations

The concentration of survivin was significantly higher in the sera of children with JIA compared to the controls (Me 23.14 pg/ml (IQR 17.37–35.31) vs Me 10.11 pg/ml (IQR 5.24–14.10); *p* < 0.001) (Fig. 1).

There was no statistically significant difference in serum survivin level between children with JIA newly diagnosed comparing to children with JIA biologically treated (Me 22.61 pg/ml (IQR 17.37–35.31) vs Me 24.72 pg/ml (IQR 19.46–40.64); *p* = 0.68).

**Fig. 2:**
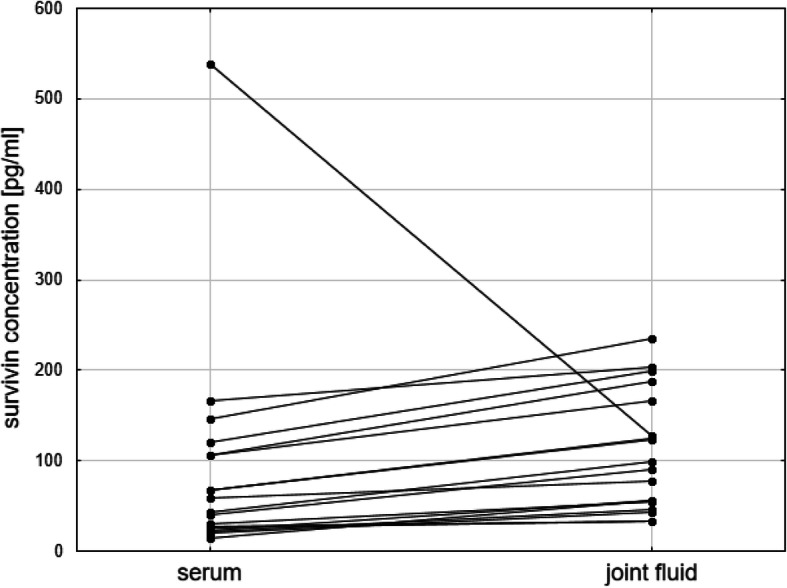
Serum and joint fluid survivin concentration (median and IQR) in children with JIA

In all but one (17/18–94.4%) synovial fluid samples, the concentration of survivin was higher than in matched serum (Me 94.15 pg/ml (IQR 54.87–165.91) vs 50.82 pg/ml (IQR 25.77–105.47); *p* = 0.003) (Fig. 2) and correlated significantly with each other (*R* = 0.88, *p* < 0.001). In that one girl with polyarticular JIA onset type lasting for 2 months and with high disease activity, survivin concentration in the serum was the highest among the whole study group (537.82 pg/ml), and the concentration of survivin in synovial fluid was above the median (127.20 pg/ml). We did not find a significant difference in synovial fluid survivin level between newly diagnosed and biologically treated children with JIA (Me = 125.59 (IQR 76.8–187.30) vs Me 56.30 (IQR 44.20–94.20); *p* = 0.143) what can be associated with a low statistical power of this comparison.

Different JIA onset types or disease duration time and disease activity according to JADAS-27 did not influence significantly survivin concentration in the serum and joint fluid. Additionally, the survivin concentration did not statistically significantly change according to radiological damage status based on hands X-ray or active synovitis grade of the affected joints assessed by joint ultrasonography (Table 2).

**Table 2:** Survivin concentration according to laboratory tests and clinical characteristics in serum (a) and synovial fluid (b)

Parameter	Groups and statistics				***P***
**(a) Serum**					
JIA onset types [medians]	1: 24.19	2: 22.61	3: 24.72		0.7956 (Kruskal-Wallis ANOVA)
Disease duration time	*R* = 0.065				0.5905 (Spearman rank correlation)
Disease activity	0: 25.24	1: 21.56	2: 22.61		0.5761 (Kruskal-Wallis ANOVA)
Radiological damage status	1: 25.77	2: 21.56	3: 22.61		0.0882 (Kruskal-Wallis ANOVA)
Synovitis activity	0: 22.61	1: 25.77	2: 21.04	3: 22.61	0.4329 (Kruskal-Wallis ANOVA)
**(B) Synovial fluid**					
JIA onset types [medians]	1: 110.97	2: 67.60	3: -		0.3736 (UMW test)
Disease duration time	*R* = −0.19				0.4430 (Spearman rank correlation)
Disease activity	0: 100.13	1: 89.87	2: 127.20		0.5134 (Kruskal-Wallis ANOVA)
Radiological damage status	1: 123.50	2: 89.87	3: 44.58		0.2211 (Kruskal-Wallis ANOVA)
Synovitis activity	0: 55.54	1: 98.43	2: 187.30	3: 125.35	0.1063 (Kruskal-Wallis ANOVA)

*IQR* interquartile range

**Fig. 3:**
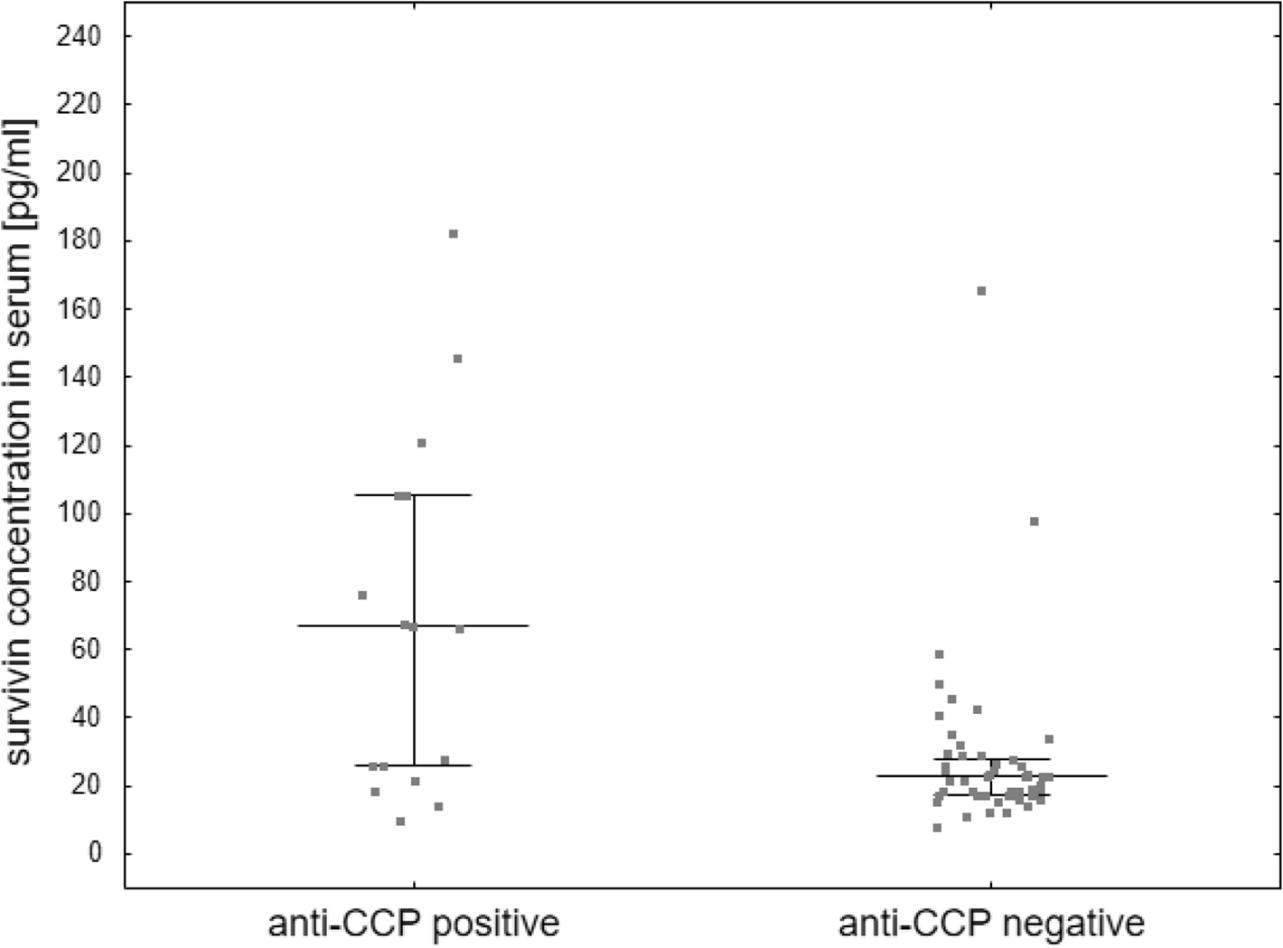
Median serum survivin concentration (median and IQR) according to anti-CCP (ACPA) positivity in children with JIA. *Patient from anti-CCP (ACPA)-positive group with a value of 537.82 pg/ml was not shown in the graph but contributed to median and IQR calculations

Statistically, higher serum survivin concentration was observed among children with JIA and the presence of anti-CCP antibodies (ACPA) in comparison with anti-CCP-negative patients (Me 66.98 pg/ml (IQR 25.77–105.47) vs Me 22.61 pg/ml (IQR 17.37–27.89); *p* = 0.001) (Fig. 3). Nonetheless, there was no significant difference between synovial fluid survivin concentration and anti-CCP (ACPA) positivity (*p* = 0.1976).

No significant difference in survivin level was observed between RF- and ANA-positive vs negative patients in neither serum nor synovial fluid of children with JIA.

**Fig. 4:**
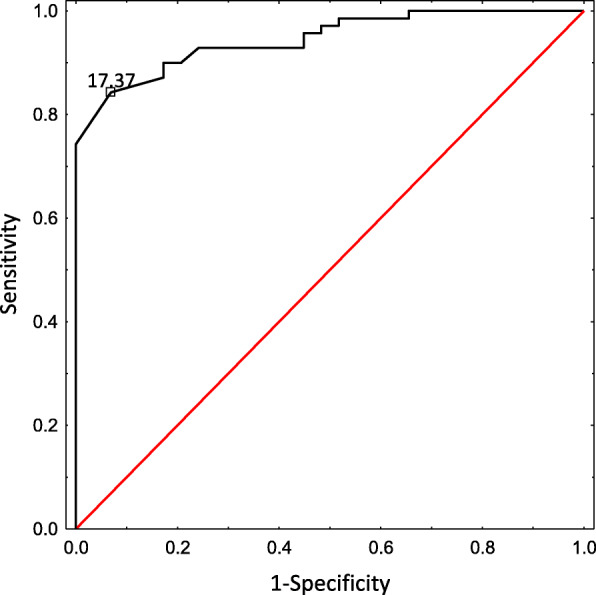
Receiver operator characteristics (ROC) analysis of survivin concentration in the sera of children with JIA (cutoff = 17.37 pg/ml)

According to the ROC analysis the cutoff for survivin concentration was calculated at 17.37 pg/ml. The area under the curve (AUC = 0.945 (95 CI 0.905–0.985)) confirmed the ability of survivin to distinguish children with JIA with a sensitivity of 0.843 (95 CI 0.732–0.915) and a specificity of 0.931 (95 CI 0.758–0.988); Youden Index value = 0.77 (Fig. 4).

According to the established cutoff value at the 17.37 pg/ml in total, 59/70 children with JIA were survivin-positive, including almost 91% (10/11) of children with JIA, who have been biologically treated. However, also 2/29 (6.9%) children from the control group were survivin-positive, but the positive concentrations in their sera were just at the cutoff level (both exactly 17.37 pg/ml).

In the group of survivin-positive children with JIA, the majority (38/59; 64.4%) represents oligoarthritis, 20/59 (33.9%) polyarthritis, and only one child had systemic JIA.

There was no statistically significant difference in survivin positivity according to gender, age, JIA onset subtype, disease duration time, disease activity, radiological damage, or ultrasonographic synovitis grade (Table 3).

**Table 3:** Clinical characteristics of children with JIA divided by *survivin* status

Variables	Survivin-positive (***N*** = 59), Median (IQR)	Survivin-negative (***N*** = 11), Median (IQR)	***p*** value
Age [years]	10 (6.5–15.5)	11.5 (5.50–17.0)	0.9227
	*N* (%)	*N* (%)	
Sex: girls	43 (72.88%)	8 (72.72%)	1.00
Disease duration time [months]	12 (1.5–36)	2 (1.5–4)	0.3721
Disease activity	0 (0–1)	0 (0–1)	0.8878
ESR [mm/h]	11 (7–30)	15 (10–20)	0.5127
CRP [mg/dl]	1 (0.31–8.93)	2.02 (0.4–12.2)	0.6806
ANA: positive	26 (44.07%)	7 (63.64%)	0.3272
RF: positive	6 (10.17%)	3 (27.27%)	0.1432
Anti-CCP (ACPA): positive	15 (25.42%)	2 (18.18%)	0.7215

Nineteen out of 59 survivin-positive children with JIA (32.2%) were also positive for RF and/or anti-CCP. Only 2 patients with positive survivin—2 (3.4%) were recognized also by the simultaneous presence of RF and anti-CCP antibodies (ACPA).

Survivin did not correlate significantly with neither CRP (*R* = − 0.07, *p* = 0.584), WBC (*R* = − 0.05, *p* = 0.696), nor ESR (*R* = − 0.20, *p* = 0.105).

## Discussion

In the current study, we demonstrate that the concentration of survivin is notably increased in the sera of children with JIA compared to healthy controls, what is in line with the previous observations in adults with RA and children with JIA [8, 16–20]. Additionally, we show that gender, age, and different JIA onset types did not influence significantly survivin concentration in the serum, as well as in joint fluid.

To our knowledge, there is only one other study assessing the survivin in the sera of children with JIA [19]. We associated the survivin level not only with laboratory data, but also with radiological status of the affected joints and with synovitis grade evaluated by ultrasonography (PDUS) of the inflamed joints. Moreover, this is the first paper that compares survivin concentration in the serum and available matched synovial fluid of children with JIA.

Previously, it was speculated that survivin is produced and secreted locally in the inflamed joints [7, 18, 21]. Here, we confirm the results obtained in adults with RA, indicating that there is a strong positive correlation between survivin level in the serum and matched synovial fluid [7, 18, 21]. Additionally, our present study demonstrates that higher survivin concentration is detected in the joint fluid than in matched serum. However, the number of available synovial fluid samples in our study was small. Nevertheless, the grade of synovitis evaluated by ultrasonography (PDUS) of the inflamed joint does not influence the survivin concentration in synovial fluid of our study group.

In the patients, early after RA diagnosis, the positivity of survivin predicts joint destruction and resistance to anti-rheumatic treatment [7–9]. Interestingly, in our research, we do not register higher survivin concentration in children with JIA and worse radiological joint destruction. This could be explained by the high number of children with newly diagnosed JIA and the fact that radiological damage in children contrary to adults is rarely observed at the early stage of the JIA, as children have the potential for bone regeneration [2, 4, 6]. Thus, we failed to confirm survivin prognostic potential for the active and destructive course of the rheumatoid process [7, 11, 21–23].

Surprisingly, we do not find a significant prior association between high survivin level and high disease activity found in adults with RA and in children with JIA [8, 11, 16, 17, 19]. Almost 63% of our study group consisted of children with oligoarticular JIA, mostly with low disease activity and short duration time of JIA symptoms. On the other side, it is confirmed that survivin level is irrespective of disease duration time. It has been postulated that rheumatoid process starts years before clinical symptoms and may be identified by autoantibody measurement. Previous studies advocated survivin to provide insight in the pre-antibody process. Some authors suggest that survivin occurs at the earlier phase of disease development followed by autoantibody production [8, 17]. Our results indicate that survivin concentration is independent of the disease duration time—could be increased in sera of newly diagnosed children at the beginning of the disease and after years, even in patients who had been biologically treated. However, some authors noticed a significant decrease in survivin levels from baseline over 2 years of follow-up [11].

The combination of various markers increases further risk of rheumatoid process and may assist its preclinical diagnosis. The other important finding of this study is the fact that anti-CCP (ACPA) positive children with JIA have an increased level of survivin in the serum comparing to ACPA-negative patients, what supports previous studies in adults with RA [8]. However, other authors did not notice the dependency of survivin presence on ACPA or RF positivity in RA patients [11]. They also underlined that neither the presence of RF or ACPA nor the combined multi-biomarker disease activity score supported discrimination in the disease outcome achieved by survivin measurements. We did not find a significant association between anti-CCP antibody positivity and higher survivin level in synovial fluid of JIA patients. The cutoff (17.37 pg/ml) established according to ROC analysis survivin positivity was found in the majority of children with JIA, including all but one child biologically treated. Thus, we confirm the ability of survivin to distinguish children with JIA with a sensitivity of 0.843 and a specificity of 0.931. The numbers are similar to that obtained by other researchers [8, 16]. However, two (6.9%) children from our control group were survivin-positive, but their serum survivin concentration was just at the cutoff value (both exactly 17.37 pg/ml). Similar percentage of the survivin positivity in the control groups received other authors—5.2–6.6% [11, 19, 21]. It could be speculated that it is possible that these children in the future could present JIA symptoms as survivin occurs before clinical manifestation, even in the pre-antibody period. As in our study group the percentage of children with JIA does not correspond to the prevalence of JIA in the general population thus PPV and NPV were not calculated.

Our results support earlier conclusions in adults and children that age, gender, JIA onset subtype, disease duration time, and presence of RF are similar in survivin-positive children with JIA, as compared with those survivin-negative [11, 19, 21]. The combined presence of survivin and autoantibodies was found in a small group of our children with JIA. One third (19/59–32.2%) of our survivin-positive study group was recognized positive for RF or ACPA, and only two children were simultaneously positive for both RF and ACPA. These results are similar to those obtained by others. Data showed an increased risk of JIA development irrespective to ACPA status. This provides further support to the hypothesis that survivin is a unique biomarker that recognizes an additional group of JIA patients with negative autoantibodies.

We assessed the survivin levels also in a group of children with JIA biologically treated. Nevertheless, that group of patients was small, and it was noticed that high survivin concentration and its positivity are independent of TNF-α treatment, which is in line with previous observations in adults with RA [7, 11, 18, 21]. On the other hand, some researchers proved that survivin concentration is decreased in TNF-α treatment adult RA responders [24, 25]. In our study, biologically treated patients with JIA were previously unsuccessfully administered with conventional synthetic DMARDs for a quite long period of time (above 1 year). Some researchers concluded that survivin could be the marker of DMARD non-responders [11, 23, 24]. However, to verify that statement in children, the group of patients with JIA treated with conventional synthetic DMARDs should be also included in the study, which is the area for future studies. Nevertheless, the observations focusing on the influence of treatment on survivin release are conflicting. The process triggering and abrogating survivin release in patients with rheumatoid process could therefore pave a way to efficient therapeutic control of the disease [11].

We are aware of the limitations of our research. First of all, there was a small control group of healthy children. To confirm the ability of survivin to differentiate JIA from other causes of joint inflammation (for example, reactive arthritis), the sufficiently large group of such patients should be also included in the future study. Additionally, our study group had a long disease duration time, with conventional synthetic DMARD treatment failure before the implementation of TNF-α inhibitors, and there is no group of children with JIA treated with DMARDs to compare. Furthermore, only two subtypes of JIA onset are properly represented, and there are a small number of joint fluid samples available. What is more, there was a small group of children with JIA biologically treated and only with TNF-α inhibitors—no group treated with other biologicals to compare. Further studies are required to establish a relationship between survivin level and apoptotic cytokine dynamics in JIA patients.

## Conclusions

On the basis of our study, it could be concluded that survivin seems to be an independent biomarker, irrespective of disease duration time, that may be helpful in the diagnosis of JIA. Nevertheless, the higher concentration of survivin is being associated with ACPA positivity, and survivin can act as a unique biomarker that identifies an additional group of patients with JIA-negative for autoantibodies even in the early stage of the disease. We have failed to confirm survivin prognostic potential for the active and destructive course of the rheumatoid process in JIA. However, it is worth to underline, that children have better potential for bone regeneration than adults. Survivin should be considered as a biomarker of joint inflammation helpful in the diagnosis of oligo- and polyarticular JIA and probably not dependent on treatment with TNF-α inhibitors.

## Data Availability

The datasets used and analyzed during the current study are available from the corresponding author on reasonable request.

## Abbreviations

ANA: Antinuclear antibodies
anti-CCP = ACPA: Anti-cyclic citrullinated peptide autoantibodies
AUC: Area under the curve
CRP: C-reactive protein
DMARDs: Disease-modifying antirheumatic drugs
ESR: Erythrocyte sedimentation ratio
ILAR: International League of Associations for Rheumatology
IQR: Interquartile range
JADAS-27: 27-Joint Juvenile Arthritis Disease Activity Score
JIA: Juvenile idiopathic arthritis
MTX: Methotrexate
PDUS: Power Doppler ultrasonographic signal
PLT: Platelets count
RA: Rheumatoid arthritis
RBC: Red blood cells
RF: Rheumatoid factor
ROC: Receiver operator characteristics
TNF-α: Tumor necrosis factor-α
WBC: White blood cells
